# Predictive Value of Monocyte Chemoattractant Protein-1 in the Development of Diastolic Dysfunction in Patients with Psoriatic Arthritis

**DOI:** 10.1155/2022/4433313

**Published:** 2022-06-03

**Authors:** Iva Uravić Bursać, Tatjana Kehler, Vedrana Drvar, Emina Babarović, Vesna Pehar Pejčinović, Antonija Ružić Baršić, Viktor Peršić, Gordana Laskarin

**Affiliations:** ^1^Department of Cardiology, Hospital for Medical Rehabilitation of Hearth and Lung Diseases and Rheumatism “Thalassotherapia-Opatija”, M. Tita 188, 51420 Opatija, Croatia; ^2^Department of Rheumatology, Hospital for Medical Rehabilitation of Hearth and Lung Diseases and Rheumatism “Thalassotherapia-Opatija”, M. Tita 188, 51420 Opatija, Croatia; ^3^Department of Medical Rehabilitation, Faculty of Medicine, University of Rijeka, B. Branchetta 20, 51000 Rijeka, Croatia; ^4^Clinical Department of Laboratory Diagnostics, Clinical Hospital Centre Rijeka, Tome Strižića 3, 51000 Rijeka, Croatia; ^5^Department of Pathology, Faculty of Medicine, University of Rijeka, B. Branchetta 20, 51000 Rijeka, Croatia; ^6^Clinical Department of Pathology, Clinical Hospital Center Rijeka, Krešimirova 42, 51000 Rijeka, Croatia; ^7^Department of Radiology, Hospital for Medical Rehabilitation of Hearth and Lung Diseases and Rheumatism “Thalassotherapia-Opatija”, M. Tita 188, 51420 Opatija, Croatia; ^8^Department of Physiology, Immunology and Pathophysiology, Faculty of Medicine, University of Rijeka, B. Branchetta 20, 51000 Rijeka, Croatia

## Abstract

We aimed to evaluate the diagnostic accuracy of the proinflammatory monocyte chemotactic protein-1 (MCP-1) in the diagnosis of asymptomatic diastolic dysfunction (DD) in patients with psoriatic arthritis (PsA). The disease activity in psoriatic arthritis (DAPSA) was determined using clinical and laboratory parameters, and echocardiography was performed to estimate DD. Serum MCP-1 concentrations were elevated in PsA patients with DD diagnosed with ultrasound (median (25^th^ percentile, 75^th^ percentile): 366.6 pg/mL (283, 407.1 pg/mL) vs. 277.5 pg/mL (223.5, 319.1 pg/mL) in controls; *P* < 0.0017). PsA patients with serum MCP-1 concentration higher than the cut-off value of 347.6 pg/mL had a 7.74-fold higher chance of developing DD than PsA patients with lower serum MCP-1 concentrations (controls), with a specificity of 86.36% and sensitivity of 55%, as verified using ultrasound. The group with MCP-1 concentrations above the cut-off value also showed a higher late peak diastolic mitral inflow velocity, A-wave value (*P* = 0.000005), E/E′ ratio (*P* = 0.00005), and a lower E/A ratio (*P* = 0.000002), peak systolic left atrial reservoir strain, SA value (*P* = 0.0066), early peak diastolic displacement of the mitral septal annulus, E′ wave value (*P* = 0.003), than controls. Systolic blood pressure (*P* = 0.01), LDL cholesterol concentration (*P* = 0.012), glucose concentration (*P* = 0.011), and DAPSA (*P* = 0.0000) increased in the PsA group with higher MCP-1 concentrations, although there were no differences in comorbidities and therapy between the groups compared. Thus, the serum MCP-1 concentration was a significant and independent prognostic indicator for asymptomatic DD in PsA patients (area under the curve = 0.730, *P* = 0.001). The DAPSA score in PsA patients might indicate the need for echocardiography and adjustment of anti-inflammatory treatment in terms of DD prevention.

## 1. Introduction

Psoriatic arthritis (PsA) is an autoimmune disease arising from the interplay between proinflammatory cytokines [1] and external stimuli in genetically predisposed individuals [2]. The disease is chronic and affects the peripheral joints and/or axial skeleton with or without extraarticular manifestations [3], including metabolic syndrome [4].

Patients with PsA show multiple cardiovascular diseases, including heart failure [5, 6]. The incidence of dilated cardiomyopathy is 10-fold higher in patients with psoriasis [7], and these patients may show asymptomatic cardiomyopathy even in the absence of traditional risk factors [8]. Many years ago, Maisch et al. [9] described this process as “inflammatory cardiomyopathy,” which first manifests echocardiographically as diastolic dysfunction (DD). Grade I DD is up to five times more common in psoriatic patients than in healthy controls [10]. The exact mechanism underlying abnormal diastolic function in patients with axial psoriatic spondyloarthritis is unclear and may depend on persistent systemic inflammation and vasculitis of the coronary arteries [11].

Monocyte chemotactic protein-1 (MCP-1) is a member of the CC chemokines, which originates from and is secreted by immune effector cells and dysfunctional endothelium [12, 13]. The pivotal function of MCP-1 is to attract monocytes into the arterial wall through increased expression of adhesion molecules on their surface that interacts with endothelium [13, 14]. MCP-1 induces the maturation of monocytes in the arterial wall, which then becomes specialized macrophages in the early atheroma. Their functions are to produce tissue factors supporting coagulation and proinflammatory cytokines such as interleukin- (IL-) 1 and IL-6. It affects the functions of the surrounding immune effector cells in the locally thickened intima [13]. MCP-1 has been shown to induce intimal and medial thickening of the human carotid arteries, which are predictors of future vascular events [15, 16]. In humans, the plasma level of MCP-1 raised with increased cardiovascular risk [14] and acute cardiovascular events [17, 18]. However, the interaction of MCP-1 and cardiovascular risk factors remains undefined.

During active disease in psoriatic skin lesions and synovial tissue, activated monocytes represent the major source of proinflammatory mediators, including the chemokine MCP-1 [19]. MCP-1 is thought to be involved in the pathogenesis of oedema and/or bone erosion in patients with PsA [5], and the MCP-1 receptor CC receptor 2 is expressed on osteoclast precursors [20]. Additionally, the plasma concentration of MCP-1 in patients with psoriasis is higher than that in healthy individuals [1]. During heart failure, MCP-1 stimulates the recruitment of proinflammatory leukocytes to the heart, cardiac fibrotic rearrangement, and dysfunction [21], since monocyte/macrophage-mediated inflammation constitutes a proinflammatory phenotype of heart failure [22]. Cardiac dysfunction is associated with a poor prognosis, increased mortality, and a high burden on society; therefore, diagnosis of cardiac dysfunction in the asymptomatic phase of the disease [23, 24] is important for the timely introduction of therapy [25].

To the best of our knowledge, no previous attempts have been made to investigate the diagnostic accuracy of MCP-1 for the determination of cardiac dysfunction. We hypothesized that MCP-1 correlates with disease activity and promotes the development of cardiovascular comorbidity in patients with PsA. This study is aimed at analyzing the relationship between serum MCP-1 concentration and echocardiographic parameters of diastolic function in asymptomatic PsA patients and investigating the diagnostic accuracy of MCP-1 in identifying the DD.

## 2. Materials and Methods

### 2.1. Patients

**Figure 1:**
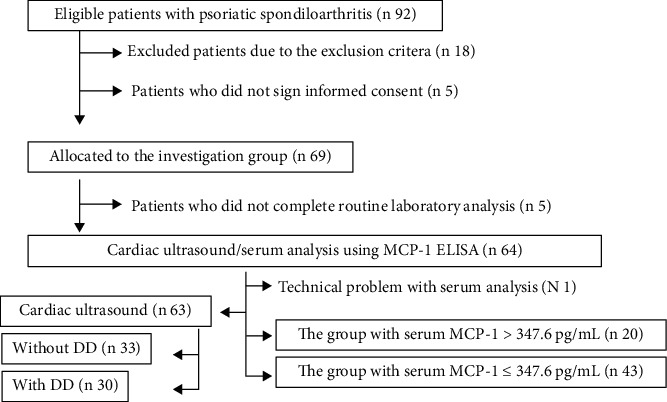
Recruitment and allocation to assessment group.

The investigation was designed as a random, prospective study. Ninety-two patients with axial and peripheral PsA without clinical manifestations of heart failure or NYHA (New York Heart Association) class 0 [26], who were diagnosed using the Assessment of Spondyloarthritis International Society (ASAS) criteria [27] and treated in rheumatologic office in “Thalassotherapia – Opatija,” Opatija, Croatia, from December 1, 2020, to December 31, 2021, were recruited in this study, according to the inclusive criterion. Eighteen patients were excluded due to the exclusion criteria, and five patients did not sign informed consent. The reminded sixty-nine patients were allocated to the investigation group. Patients who did not complete routine laboratory analyses (*n* 5) and serum MCP-1 analysis due to technical problems were also omitted from the study. Cardiac ultrasound and serum MCP-1 were done on 63 patients. The recruitment and allocation to the assessment group is shown the STARD flow diagram (Figure 1).

At the rheumatological examination disease activity was assessed using the Bath Ankylosing Spondylitis Disease Activity Index (BASDAI) for the axial skeleton [28] and the disease activity index in psoriatic arthritis (DAPSA) for peripheral arthritis [29]. The functional status of the patients was assessed using the Bath Ankylosing Spondylitis Disease Functional Index (BASFI) [30], and the spread of psoriasis was assessed using the body surface area (BSA) method [31]. On examination by a cardiologist, body height and weight were measured, body mass index (BMI) was calculated, and arterial blood pressure was measured using a sphygmomanometer (Rossmax, Heerbrugg, Switzerland). Comorbidities and medications were recorded from the medical records.

Exclusion criteria were as follows: infection, bone marrow disorders, blood transfusions, immune deficiency, uncontrolled blood glucose concentration (plasma glucose > 11 mmol/L), uncontrolled hypertension (systolic > 180 mmHg or diastolic > 110 mmHg), chronic liver or renal failure, injury to organs, or a malignant disease within 5 years.

The patients provided consent to participate in the study, which was approved by the Ethics Committees of the Hospital “Thallassotherapia – Opatija,” no. 01-000-00-751/2-2020, from December 1, 2020, and the Faculty of Medicine, University of Rijeka, no. 003-08/21-01/34 according to the “Ethical principles for medical research involving human subjects” in the Declaration of Helsinki (1964) outlined by the World Medical Association.

### 2.2. Serum Analyses

The patient's peripheral blood was sampled once (6 mL), and the serum was set aside. The concentrations of C-reactive protein (CRP), glucose, low-density lipoprotein (LDL) cholesterol, and amino-terminal probrain natriuretic peptide (NT-pro-BNP) were determined in serum by using an automatic Biochemical Analyzer Dimension Xpand (Siemens Healthcare Diagnostics, Newark, DE, USA). A part of the serum was aliquoted and stored at -20°C until the MCP-1 concentration was analyzed using the Human MCP-1 kit (Abcam, Cambridge, UK; sensitivity, 1.26 pg/mL, ab179886) and the enzyme-linked immunosorbent assay (ELISA) method with samples diluted 1 : 5, in accordance with the manufacturer's instructions. The absorbance was measured at 450 nm by using Euroimmun Analyzer I-2P (Euroimmun Medizinische Labordiagnostika AG, Lübeck, Germany) and CurveExpert Version 1.40 (Copyright © 1995-2009 by Daniel G. Hyams, Hyams Development).

### 2.3. Determination of Myocardial Damage by Echocardiography

All participants underwent transthoracic echocardiography according to the current recommendations of the American Society of Echocardiography and the European Association of Cardiovascular Imaging [32, 33]. The echocardiographic system GE Vivid 9 (General Electric Company, New York, USA) and standard apical two-, three-, and four-chamber view sections with standard measurements (2D echocardiography and Doppler echocardiography) were used for evaluation. For all patients with PsA, we evaluated the correlation of the serum MCP-1 concentration with diastolic parameters at the level of the mitral inflow (early peak diastolic mitral inflow velocity (E-wave), late peak diastolic mitral inflow velocity (A-wave), E/A ratio, left atrial volume indexed to the BSA (LAVI), and maximal velocity of tricuspid regurgitation (TR)), tissue Doppler imaging parameters (peak early diastolic displacement of the mitral septal annulus (E′) and E/E′ ratio), and measures of deformation of the left atrium (peak systolic left atrial reservoir strain (SA), peak strain rate of the left atrium conduit phase (SrE), and peak strain rate of left atrium contractile phase (SrA)) as well as parameters of systolic function of the left ventricle (ejection fraction (EF) and global systolic longitudinal strain (GLS)).

Diastolic function was analyzed by evaluating parameters of mitral inflow, tissue Doppler imaging (TDI) findings, and left atrial deformity. The parameters of mitral inflow were analyzed by measuring the E and A waves in apical four-chamber pulse Doppler imaging at the tips of the mitral valve cusp and calculating the E/A ratio. TR was measured from the apical four-chamber view as the maximal velocity of tricuspid regurgitation. The two-dimensional (2D) volume of the left atrium was calculated using the biplane method of discs, which was then indexed to the BSA. The TDI parameter of the rate of the E ҆ wave was measured from the apical four-chamber, and the E/E′ ratio was calculated. Diastolic function was labeled as normal (absent, no DD) or abnormal (DD present; abnormal relaxation (grade I), pseudonormal (grade II), restrictive (grade III)). Deformation measurement analysis was performed by 2D speckle tracking using Echo Pac software (GE Healthcare, USA). At least three cardiac cycles were recorded in the expiratory phase in the left-lateral position with continuous echocardiographic (ECG) monitoring. The LA endocardial border was manually traced at the end-systole in the apical four-chamber view. SA was measured from the maximal inflection point on the LA strain curve generated by the software system. SrE and SrA were measured from the software system as the maximal negative inflection points on the LA strain curve. Analysis of GLS of the left ventricle was performed in the apical three, four, and two views as the mean value. The systolic functions of the left ventricle were measured using the biplane Simpson method. All data were stored on a PC workstation and analyzed using EchoPac (GE Healthcare, USA).

### 2.4. Statistical Analyses

According to the preliminary serum MCP-1 concentrations in PsA patient, we calculated the sample size (*n* = 61) for the *T*-test of independent samples at a level of statistical significance *P* < 0.05 with a statistical analysis power of 90% using options “Power analysis” and “Sample size analysis” in Statistica 14.0.0.15 (TIBCO, Software Inc., PaloAlto, California, SAD). All the other statistical analyses were performed using MedCalc for Windows, version 20.011 (MedCalc Statistical Software bvba, Ostend, Belgium).

Due to the sample size, MCP-1 concentrations, and some cardiac function parameters that deviated significantly from the normal data distribution (*P* < 0.05) when evaluated by D'Agostino Pearson test, nonparametric tests were used for further evaluation of the data.

The correlations between the MCP-1 concentration and cardiac function parameters were calculated using the Spearman's rank correlation analysis. Differences between categorical variables were analyzed using *χ*^2^ test, whereas The Mann–Whitney *U* test was used for an assessment of differences between continuous variables. All tests were two-tailed, and statistical significance was set at *P* < 0.05. Continuous variables are presented as median and percentiles. Categorical variables are presented as the number of cases. A receiver operating characteristic (ROC) curve was generated for MCP-1 serum concentrations to assess the ability of this biomarker as an indicator of DD and to create the optimal statistical cut-off values. Therefore, the ROC curve and Youden index were calculated to maximize the sensitivity and specificity of the individual marker of DD in the univariate model. The area under the ROC curve (AUC) for the score model with 95% confidence interval (CI) was measured. We wanted to analyze if serum MCP-1 concentration can predict the occurrence of DD, so we performed the method of logistic regression to compute the odds ratio (OR) for predictors of the outcome (DD). The sensitivity, specificity, positive and negative likelihood ratios, and positive and negative predictive values were calculated for MCP-1 to determine its potential to diagnose DD.

## 3. Results

### 3.1. Interrelationship of MCP-1 and Parameters of Cardiac Function

**Figure 2:**
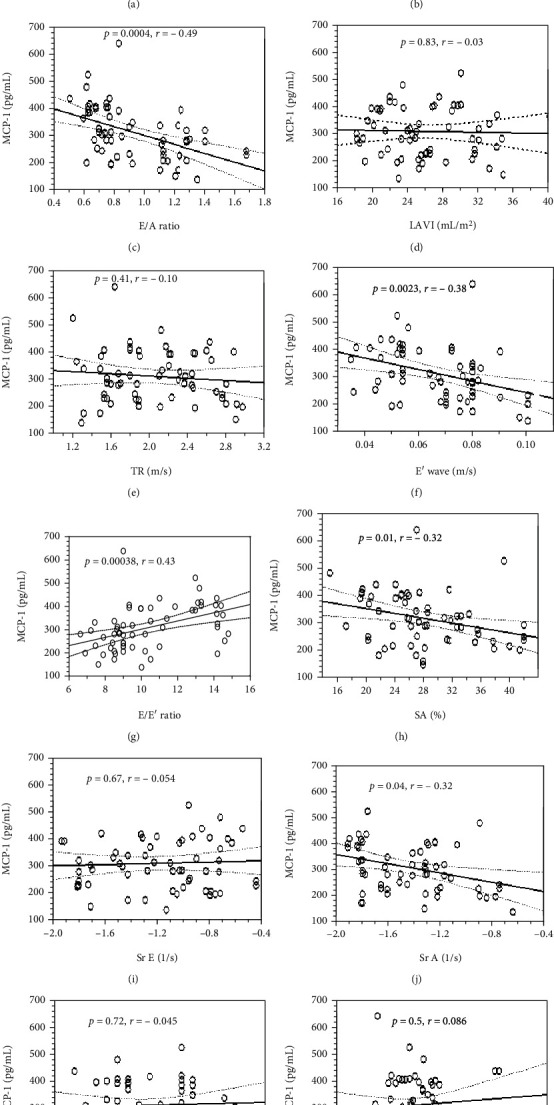
Relationship of serum MCP-1 concentration with the E-wave (a), A-wave (b), E/A ratio (c), indexed left atrial volume to the body surface, LAVI (d), the maximal velocity of tricuspid regurgitation, TR (e), E′ wave (f), E/E′ ratio (g), SA—peak systolic left atrial reservoir strain (h), SrE—peak strain rate of the left atrium conduit phase (i), SrA—peak strain rate of the left atrial contractile phase (j), ejection fraction (EF) (k), and global longitudinal strain (GLS) (l). The levels of statistical significance (*P*) and correlation coefficients (*r*) are shown in graphs.

We evaluated the correlation of the serum MCP-1 concentration and diastolic parameters in all patients with PsA. The serum MCP-1 concentration in patients with PsA did not correlate with the E-wave (Figure 2(a)), whereas it showed a significant positive correlation with the A-wave (*P* = 0.00001, Figure 2(b)). The MCP-1 concentration was negatively correlated with the E/A ratio (*P* = 0.00004, Figure 2(c)). However, it did not show a correlation with the LAVI (Figure 2(d)) or TR (Figure 2(e)) values. The MCP-1 concentration showed a significant negative correlation with the TDI parameters E′ wave (*P* = 0.0023, Figure 2(f)), whereas the correlation between the MCP-1 concentration and the E/E′ ratio was positive (*P* = 0.00038, Figure 2(g)).

We also investigated the correlation of the MCP-1 concentration with parameters of atrial deformity in patients with PsA. The MCP-1 concentration showed a significant negative correlation with the SA value (*P* = 0.01, Figure 2(h)) and the SrA waves (*P* = 0.04, Figure 2(j)), whereas it was not correlated with the SrE wave (Figure 2(i)). MCP-1 concentrations did not correlate with the systolic function parameters EF (Figure 2(k)) and GLS (Figure 2(l)).

### 3.2. Diagnostic Accuracy of MCP-1 for the Diagnosis of Asymptomatic Isolated DD

**Figure 3:**
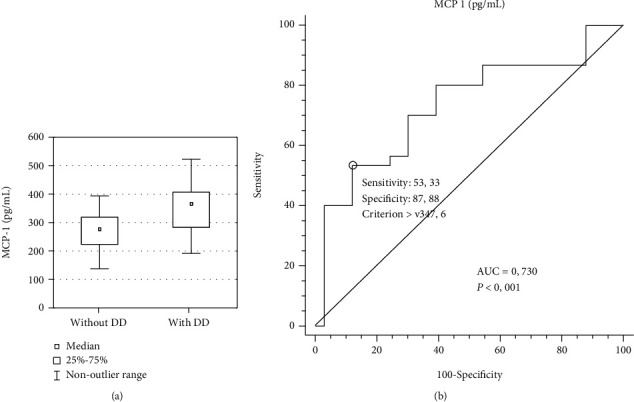
The serum concentration of MCP-1 shows differences between PsA patients without diastolic dysfunction (DD) and with DD (grade 1 and grade 2) (a), and the area under the receiver operating characteristics curve (AUC) for MCP-1 concentration at the optimal cut-off value of 347.6 pg/mL for the diagnosis DD in PsA patients (*n* = 20) with respect to PsA patients without DD (*n* = 43). Levels of statistical significance obtained in the Mann–Whitney test (a) and receiver operating characteristic analysis (b) are indicated in the plots.

Among the patients with PsA (*n* = 63), 30 (47.61%) showed DD, as diagnosed by echocardiography. These patients showed significantly higher serum MCP-1 concentrations (median (25^th^; 75^th^ percentiles), 366.6 pg/mL (283, 407.1 pg/mL)) relative to the concentrations in PsA patients without DD (*n* = 33; 277.5 pg/mL (223.5, 319.1 pg/mL); *P* = 0.0017; Figure 3(a)). This prompted us to perform ROC curve analysis which indicated that a serum MCP-1 concentration of 347.6 pg/mL was the cut-off value for DD in PsA patients (Figure 3(b)). The findings indicated that the MCP-1 concentration was a significant and independent prognostic indicator of DD in PsA patients, with a specificity of 86.36% and sensitivity of 55% (Figure 3(b)).

Based on the cut-off value for the serum MCP-1 concentration, the patients were classified into two groups: (a) the group with serum MCP-1 concentration higher than the cut-off value (*n* = 20) and (b) the group with the serum MCP-1 concentrations equal to or lower than the cut-off value (*n* = 43), which served as the controls. Patients with PsA who had a serum MCP-1 concentration higher than 347.6 pg/mL had a 7.74-fold higher chance of developing DD than controls (Figure 3(b)). The positive predictive value of the serum MCP-1 concentration was 80.0% (95% CI, 60.1% to 91.4%), and the negative predictive value was 67.4% (95% CI, 58.1% to 75.6%).

### 3.3. Differences in PsA Patients with respect to the MCP-1 Concentration

Table 1 shows a comparison of echocardiographic parameters of patients with PsA classified into dichotomous groups according to the cut-off value of serum MCP-1 concentration (347.6 pg/mL). The E-wave did not significantly differ between dichotomous groups, and the E/A ratio was lower in the group with higher MCP-1 concentrations (*P* = 0.000002) due to the higher value of the A-wave (*P* = 0.000005). The LAVI and TR values did not differ between the groups. The E/E′ ratio was higher in the group with higher MCP-1 concentrations (*P* = 0.00005), corresponding to a lower E′ wave value (*P* = 0.003). SA was lower in the group with higher MCP-1 concentrations (*P* = 0.0066), whereas SrA and SrE did not differ between the groups. Systolic function, EF, and GLS did not differ between the groups.

Table 2 lists the clinical and laboratory characteristics of patients. Systolic blood pressure (*P* = 0.01), LDL cholesterol concentration (*P* = 0.012), glucose (*P* = 0.01) concentration, and DAPSA (*P* = 0.0000) were higher in the PsA group with higher MCP-1 concentrations than in the controls. The age, sex, BMI, BASDAI, BASFI, BSA, and laboratory parameters (CRP and NT-pro-BNP) did not differ significantly between the groups. The frequency of comorbidities (arterial hypertension, diabetes, and cigarette smoking) and treatment with nonsteroid antirheumatics, synthetic (s) disease-modifying antirheumatic drugs (DMARDs), and tumor necrosis factor (TNF) inhibitors as biologic DMARDs, angiotensin-converting enzyme inhibitors, and beta-blockers were basically the same in both groups. In the group with lower MCP-1 concentrations, five subjects were taking dual antihypertensive therapy (angiotensin-converting enzyme inhibitor and beta-blocker), and in seven patients, arterial hypertension was newly detected on examination. In the group with higher MCP-1 concentration, seven patients received dual antihypertensive therapy, and five patients had newly diagnosed arterial hypertension.

## 4. Discussion

The ability to distinguish patients aged <60 years with asymptomatic DD from a very large proportion of PsA patients who often express classical cardiovascular risk factors still represents a diagnostic challenge owing to the lack of highly specific and sensitive biomarker(s) [34]. The present study is the first to indicate that MCP-1 can serve as a noninvasive, independent prognostic parameter to diagnose isolated asymptomatic DD in patients with PsA and preserved left ventricular EF ≥ 50, as confirmed by echocardiography, without any adverse events from performing the tests. NT-pro-BNP concentrations did not differ between the PsA patients, although it has been reported to be a marker of DD in symptomatic patients, as determined by invasive measurements [34, 35]. In a previous study, the NT-pro-BNP concentration was not elevated in the plasma of approximately 20% of patients with grade I DD [35], similar to the findings for most PsA patients in this investigation. In contrast, the serum MCP-1 concentration was significantly higher in the PsA group with asymptomatic DD in comparison with the concentration in patients without DD. Higher MCP-1 concentrations were associated with the increased risk particularly of ischemic stroke, in dose-dependent manner, independently of age, sex, race, and vascular risk factors [36]. Similarly, multivariable linear regression analysis confirmed that serum MCP-1 concentration was an independent predictor of asymptomatic DD in PsA patients, with a comparable to that of plasma NT-pro-BNP for symptomatic DD [35, 37]. The sensitivity of the MCP-1 concentration (55%) was lower than that of NT-pro-BNP [35], whereas it was similar to that of end-diastolic pressure, which has a sensitivity of 61% and represents the most specific ultrasound parameter for symptomatic isolated DD [35]. DD attenuates spontaneous mitral inflow measured by pulsed Doppler in the early diastole, which is thought to be compensated by atrial contraction in the late diastolic phase [38]. This is manifested by a decrease in the E-wave velocity and an increase in the A-wave velocity, which are early signs in patients with plaque psoriasis [39] and PsA [40]. Accordingly, in PsA patients with higher serum MCP-1 concentrations, the E/A ratio was significantly lower due to the significantly higher value of the A-wave. LAVI and TR, the markers of symptomatic advanced DD [37], did not correlate with MCP-1 concentration nor did they differ between the two groups of PsA patients, since ultrasound changes in PsA patients were in favour of initial DD. Moreover, some inaccuracies have been reported in the measurement of LAVI [41, 42] and TR [43].

Serum MCP-1 concentrations showed a negative correlation with the E′ wave in asymptomatic DD, as did NT-pro-BNP concentrations in symptomatic DD [35], whereas they were positively correlated with the E/E′ ratio in PsA patients, as measured with TDI. An increase in the E/E′ ratio corresponds to deterioration of the diastolic functional grade [44]. The E/E′ ratio was significantly higher in PsA patients with higher MCP-1 concentrations, corresponding to a lower E′ wave value, than in patients with low MCP-1 concentrations. Thus, the MCP-1 concentration precisely reflects the parameters associated with DD and myocardial damage [45], since TDI is less dependent on age and volume load than Doppler echocardiography [33].

A prolonged increase in left ventricular filling pressures results in dilatation of the left atrium [44, 46]. The maximum volume of the left atrium affects the outcomes of cardiovascular disease and can be used to stratify the risk of death from heart failure [47]. In comparison with TDI, measurements of left atrial deformation and atrial function as a reservoir are more precise predictors of the degree of DD and left ventricular diastolic pressure [47–49], as well as the degree of atrial deformation over a unit of time. The advantage of deformation techniques over TDI is that they are independent of the angle of isonance, can be measured overall walls of the left atrium [49], and are less dependent on preload [50]. We showed that in PsA patients, the MCP-1 concentration was negatively correlated with SA, indicating that in psoriatic “inflammatory cardiomyopathy,” the atria lose their reservoir function. A gradual decrease in SA appears as the diastolic functional grade worsens or E/E′ increases [41, 44]. Additionally, patients with higher MCP-1 concentrations had significantly lower SA levels than the controls. Left ventricular failure is associated with SA < 24% regardless of left atrial volume in asymptomatic patients older than 70 years [25]. A decrease in SA, regardless of age, sex, left atrial volume, and heart rate, has also been demonstrated as a risk factor for heart failure in patients with arterial hypertension [51].

The MCP-1 concentration showed a significant negative correlation with the SrA and indicated that a lower degree of the atrial deformity was associated with an increased serum MCP-1 concentration in patients with PsA. The systolic function parameters EF and GLS were not associated with serum MCP-1 concentration, indicating that MCP-1 is a marker of asymptomatic DD. However, MCP-1 is not determined in routine laboratory praxis, which limits its use to a larger number of PsA patients. This also hinders the confirmation of these results in a larger sample of patients with PsA, worldwide.

Patients classified into groups with respect to MCP-1 concentrations also showed different clinical and laboratory characteristics. In this study, systolic blood pressure and LDL cholesterol concentrations, as well as the DAPSA score, were significantly higher in PsA patients with MCP-1 concentration above the serum cut-off value than in the controls. DAPSA is a measure of peripheral PsA activity, which reflects the synovial inflammatory response and includes parameters such as the CRP concentration, tender and swollen joint counts, and patient assessment of disease activity and pain [29]. During periods of pain, there is a greater abundance of proinflammatory mediators [52], which are released into the circulation from the inflamed joints, where they might stimulate innate and adaptive immunity and serve as biological markers of inflammation [53]. Th-1 and Th-17 effectors [54] and MCP-1 [5, 20], which are characteristic of PsA, participate in inflammation and pain, lead to increased blood pressure [55, 56] and support atherogenesis, especially in patients with metabolic syndrome [54, 57], and stimulate organ damage [55]. Increased blood pressure in PsA patients with higher serum MCP-1 concentrations in this investigation confirmed that inflammatory and metabolic factors synergize with atherogenesis without mutual exclusion [57].

Inflammation has been recognized as a major pathophysiological contributor to the entire spectrum of human heart failures [58], and treating PsA would also prevent the onset of DD. In this investigation, there are no statistical differences in the frequencies of patients using the main groups of antirheumatic drugs. However, none of the patients did use treatment with anti-IL-17 antibodies, which might diminish MCP-1 production according to the newest comprehension [59], which represents the weakness of the study.

In conclusion, a high serum concentration of MCP-1 in PsA patients increases the probability of DD diagnosis by 7.74-fold, which is confirmed by echocardiographic evaluation at the level of mitral inflow, TDI, and atrial deformation techniques. In addition to verification of DD by cardiac ultrasound and determination of serum MCP-1 concentration, the DAPSA score in PsA patients may indicate the need for adjustment of anti-inflammatory treatment in terms of prevention of DD.

## Figures and Tables

**Table 1:** Comparison of echocardiographic parameters in patients with PsA classified into groups with respect to the cut-off MCP-1 concentration (347.6 pg/mL).

	Patients with serum MCP-1		*P* value
	≤347.6 pg/mL (*n* = 43) Median (25^th^; 75^th^ percentiles)	>347.6 pg/mL (*n* = 20) Median (25^th^; 75^th^ percentiles)	
E wave (m/s)	0.7 (0.62; 0.78)	0.67 (0.63; 0.71)	0.452
A wave (m/s)	0.62 (0.56; 0.75)	0.99 (0.86; 1.08)	0.000005∗
E/A ratio	1.11(0.78; 1.25)	0.68 (0.63; 0.77)	0.000002∗
LAVI (ml/m^2^)	25.25 (22.92; 29.93)	26.9 (21.99; 29.32)	0.844
TR velocity (m/s)	1.99 (1.57; 2.45)	2.01 (1.76; 2.46)	0.747
E′ (m/s)	0.076 (0.066; 0.08)	0.05 (0.049; 0.063)	0.003∗
E/E′ ratio	9 (8.5; 10.2)	12.98 (10.36; 13.5)	0.00005∗
SA (%)	27.6 (25.44; 33.4)	23.5 (19.9; 25.04)	0.0066∗
Sr E (1/s)	-1.22 (-1.61; -0.92)	-1.02 (-1.31; -0.78)	0.228
Sr A (1/s)	-1.41 (-1.65; -1.21)	-1.75 (-1.8; -1.3)	0.09
EF (%)	55 (55; 60)	55 (54; 60)	0.46
GLS (%)	-21(-22.6; -19.7)	-20.3 (-21.8; -19)	0.37

MCP-1: Monocyte Chemoattractant Protein-1; E: early peak diastolic mitral inflow velocity; A: late peak diastolic mitral inflow velocity; LAVI: left atrial volume indexed to the body surface area; TR: maximal velocity of tricuspid regurgitation; E′: early peak diastolic displacement of the mitral septal annulus; SA: peak systolic left atrial reservoir strain; Sr E: peak strain rate of left atrium conduit phase; SrA: peak strain rate of left atrium contractile phase; EF: ejection fraction; GLS: global longitudinal strain. ∗Level of *P* value with statistical significance, Mann–Whitney test.

**Table 2:** Clinical and laboratory characteristics of PsA patients classified into groups with respect to the cut-off MCP-1 concentration (347.6 pg/mL).

	Patients with serum MCP-1		*P* value
	≤347.6 pg/mL (*n* = 43) Median 25^th^; 75^th^ percentiles	>347.6 pg/mL (*n* = 20) Median 25^th^; 75^th^ percentiles	
Clinical characteristics and laboratory parameters			
BASDAI	3.1 (1.05; 3.94)	4 (2.7; 4.95)	0.236^a^
BASFI	1.75 (1.1; 2.8)	2 (1.35; 5.6)	0.19^a^
DAPSA	10.6 (8.3; 18.5)	18.45 (11.77; 32.35)	0.0000^a^
BSA (%)	0.25 (0; 1)	0 (0; 2)	0.572^a^
BMI (kg/m^2^)	26.9 (25.65; 29.9)	30.3 (27.15; 33.8)	0.082^a^
Diastolic pressure (mmHg)	85 (77.25; 92.75)	89.5 (80; 100)	0.1^a^
Systolic pressure (mmHg)	129.5 (102; 180)	150 (128.75; 159.25)	0.01^a^
CRP (mg/L)	1.2 (0.8; 2.8)	1.65 (0.95; 9.1)	0.249^a^
Glucose (mmol/L)	5.2 (4.9; 5.65)	5.8 (5.3; 6.5)	0.011^a^
LDL-cholesterol (mmol/L)	3.7 (3; 4.1)	4 (3.57; 5.17)	0.012^a^
NT-pro-BNP (pmol/L)	60 (35; 136)	57 (42; 62.25)	0.803^a^
MCP-1 (pg/L)	267.7 (214.2; 299.2)	404.1 (391; 422.9)	0.00063^a^
Age (year)	61 (54; 64)	62 (55.75; 67.25)	0.478^a^
Comorbidities and current therapy (*n*)			
Type II diabetes	4	3	0.503^b^
Arterial hypertension	21	14	0.115^b^
Cigarette smoking	7	6	0.210^b^
sDMARD	13	7	0.705^b^
TNF inhibitors	13	10	0.129^b^
NSAR	17	3	0.051^b^
ACE inhibitors	8	7	0.155^b^
Beta blockers	11	9	0.123^b^

MCP-1: Monocyte Chemoattractant Protein-1; BASDAI: Bath Ankylosing Spondylitis Disease Activity Index; BASFI: Bath Ankylosing Spondylitis Functional Index; DAPSA: Disease Activity in Psoriatic Arthritis; BSA: body surface area; BMI: body mass index; NYHA: New York Heart Association Classification; LDL: low-density lipoprotein; NT-pro-BNP: N-terminal probrain natriuretic peptide; sDMARD: synthetic disease-modifying antirheumatic drugs; TNF: tumor necrosis factor; NSAR: nonsteroid antirheumatics; ACE: angiotensin-converting enzyme. Levels of statistical significance are indicated as *P* values in ^a^Mann–Whitney test; ^b^Chi-squared test.

## Data Availability

The data sets used and/or analyzed during the present study are available from the corresponding author on reasonable request.
